# Could VGF and/or its derived peptide act as biomarkers for the diagnosis of neurodegenerative diseases: A systematic review

**DOI:** 10.3389/fendo.2022.1032192

**Published:** 2022-12-22

**Authors:** Saleha Alqarni, Mashael Alsebai

**Affiliations:** Division of Nutritional Sciences, School of Biosciences, University of Nottingham, Nottingham, United Kingdom

**Keywords:** VGF, VGF peptides, neurodegenerative diseases, amyotrophic lateral sclerosis, Alzheimer’s disease, Parkinson’s disease

## Abstract

**Background:**

The increasing ageing population has led to an increase in the prevalence of neurodegenerative diseases, such as Alzheimer’s disease (AD), Parkinson’s disease (PD), and amyotrophic lateral sclerosis (ALS). However, as yet, there are no simple biomarkers to predict the onset of such diseases. Recently, VGF and its peptides have been highlighted in neurodegenerative diseases. VGF (non-acronymic) is a polypeptide induced in PC12 cells by neurotrophic factors.

**Objective:**

This systematic review aimed to determine whether VGF and/or its derived peptides can be used as biomarkers for the diagnosis of ALS, PD, and AD with specific attention to (1) the levels of VGF and/or its derived peptides, (2) amyloid-beta, (3) dopamine, and (4) cognitive score.

**Methodology:**

A search was undertaken in the Ovid EMBASE, Cochrane Library, PubMed, Scopus, and Web of Science for observational studies. Publications that assessed the level of VGF and/or its derived peptides among people with neurodegenerative diseases and compared them with healthy people were included. The quality of the included studies was assessed using the National Heart, Lung, and Blood Institute Quality Assessment Tool.

**Result:**

A search of the databases yielded 834 studies, of which, eight observational studies met the inclusion criteria with a total of 673 participants (51.7% males) aged >18 years. Seven studies showed significant decreases in VGF and its derived peptides in adults with AD, PD, and ALS compared to healthy controls (*p*<0.05). However, one study showed that there was no significant difference in VGF in AD compared to healthy control(*p*>0.05). Furthermore, only one study reported that VGF levels were positively correlated with those of tissue dopamine but not with Aβ1-42, and low levels of VGF were associated to cognitive deficits.

**Conclusion:**

The use of VGF and its derivatives for the diagnosis of PD, ALS, AD remains unclear, so further investigation of the role of VGF in neurodegenerative diseases and pathophysiology is needed to provide new insights.

## Introduction

Neurodegenerative diseases are becoming more common, including Alzheimer’s disease (AD), Parkinson’s disease (PD), and amyotrophic lateral sclerosis (ALS) (1), partly due to changes in the human life cycle, as the average lifespan of the population has increased (2). In 1901, the average life expectancy of men and women at birth was 48.5 and 52.4 years respectively (3) but this has increased to 79.4 years for men and 82.1 years for women in the United Kingdom between 2017 and 2019 (4). Furthermore, the proportion of individuals aged 65 years and over increased by 3.8% between 1976 and 2016 (5). By 2046, the ageing population is expected to account for about one-quarter of the population (5). Consequently, the increasing ageing population is a global phenomenon significantly impacting the quality of life as well as healthcare provision of people with neurodegenerative diseases, such as AD, PD, and ALS (6, 7). However, there are no simple biomarkers that can predict the onset of these diseases.

Recently, VGF and its peptides have been highlighted as potential biomarkers for the diagnosis of AD, PD and ALS. VGF is a polypeptide that is rapidly induced in pheochromocytoma cells (PC12 cells) treated with nerve growth factor (NGF) (8). Its unabbreviated name is based on the selection of this clone from plate V of a PC12 cell library (cDNA) induced by NGF (8). It was first identified as VGF8a (8), a2 (9) and NGF33.1 (10). Subsequent experiments showed that VGF is also regulated by various neurotrophic factors, such as brain-derived neurotrophic factor (BDNF) (11), and VGF transcription is accompanied by translation in hippocampal cultures within three hours of BDNF treatment. In addition to neurotrophic factors, VGF mRNA responds to seasonal rhythms (12), fasting (13), and salt loading (14).

VGF mRNA is found in the hypothalamus, cerebellum, main and accessory olfactory bulbs, hippocampus, cortex thalamus, and brainstem (15), as well as in endocrine tissues, such as the pituitary gland, adrenal glands, and pancreas, and the myenteric plexus and endocrine cells in the gastrointestinal tract (15, 16). VGF mRNA is upregulated by learning, neuronal activity, and lesions (17), and decreased in aged-impaired rats (17), as well as among drug-free depressed individuals (18).

**Figure 1:**
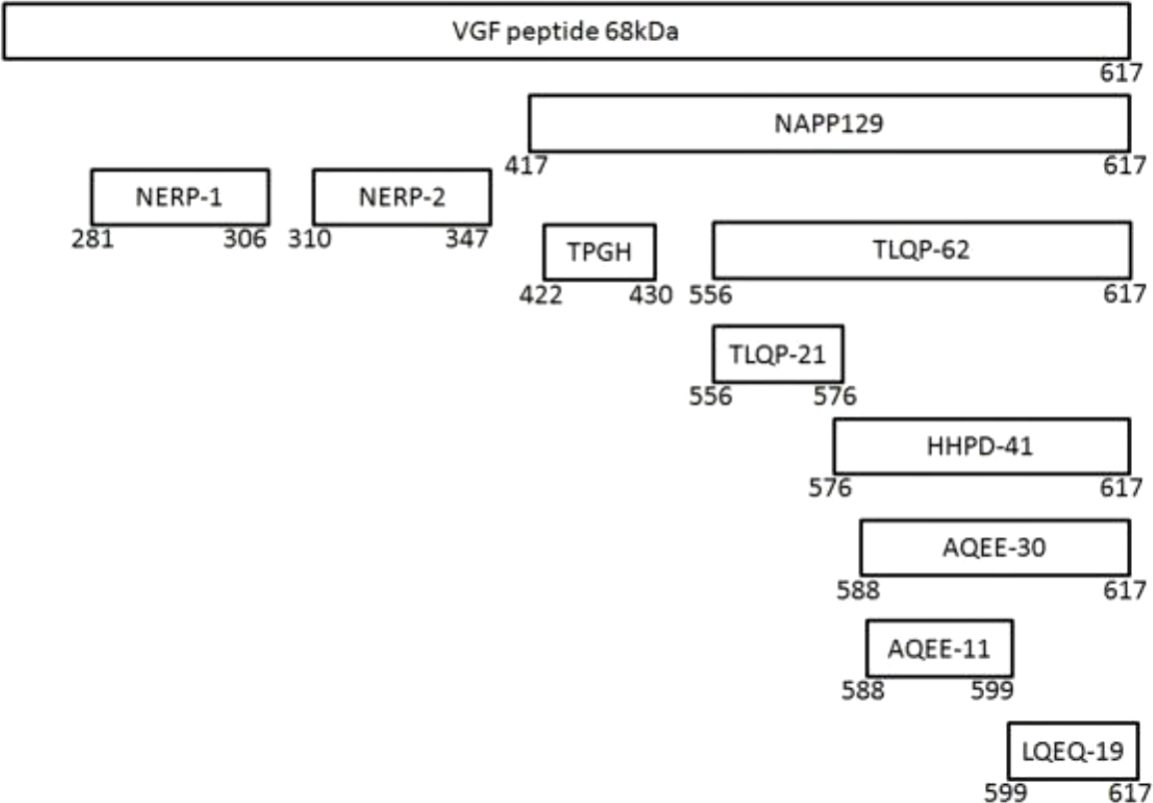
The VGF and its derived peptides.

The coding region of the *vgf* gene, as well as the promoter and flanking 5’ regulatory sequence, are highly conserved across numerous mammalian species (19). The *vgf* gene produces a polypeptide of 615 amino acids in humans and 617 amino acids in rats and mice, with a secretory leader sequence of twenty-two amino acids that promotes endoplasmic reticulum translocation (16). Several peptides have been identified in the proteolytic processing of VGF, including VGF20 or NAPP129 and VGF10 or TLQP-62 (10, 20–22), and many other peptides shown by proteomic studies (**Figure 1**). The VGF-derived peptides were identified and named by their first 4 N-terminal amino acids and their total length (23), e.g., VGF_281–306_ (NSEP-1). The cleavage sites in VGF _1-615_ can be used to classify peptides produced from VGF. The method for identifying VGF-derived peptides, their function, and the cleavage sites are shown in **Table 1**.

**Table 1:** Peptide identification method, cleavage sites, and function.

Reference	VGF-derived peptide	Method for identification of VGF-derived peptide	Protease& cleavage sites for human VGF	Function of VGF-derived peptide
**(** 24 **,** 25 **)**	NERP-1	Immunohistochemistry in temporal, parietal, and frontal cortex	R280-R281 (PC1/3 or PC2); A306-G307	Fluid homeostasis by regulating vasopressin release
**(** 24 **-** 26 **)**	NERP-2	mass spectrometry analysis and radioimmunoassay analysis in human medullary thyroid carcinoma and rat brains	R309-Q310 (PC1/3 or PC2); G347-G348	Fluid homeostasis by regulating vasopressin release
**(** 27 **)**	NAPP-129	mass spectrometry in plasma and Gel chromatography	K484-N485 (PC1/3 or PC2)	it is in neuronal and islet cell secretory granules and released in response to stimulation
**(** 11 **,** 28 **-** 30 **)**	TLQP-62	Immunohistochemistry in temporal, parietal, and frontal cortex	R553-T554 (PC1/3)	transient potentiation in rat and mouse hippocampal slices, increases synaptic activity, influences cognitive pathways, stimulates neurogenesis
**(** 31 **,** 32 **)**	TPGH	Immunohistochemistry in temporal, parietal, and frontal cortex	K418-R419 (PC1/3 or PC2); H427-R428 (PC1/3 or PC2)	It is expressed in many areas of the brain as well as in ghrelin cells in the stomach, but neither fasting nor feeding status had an effect on this.
**(** 12, 20 **,** 33 **)**	TLQP-21	gel chromatography and mass spectrometry in plasma; Immunohistochemistry in temporal, parietal, and frontal cortex	R553-T554 (PC1/3); R574-H575	regulates energy balance gastrointestinal contractile activity
**(** 21, 34 **)**	AQEE-11	matrix assisted laser desorption ionization-time of flight-mass spectrometry in rat brain	R585-A586 (PC1/3 or PC2); R596-L597 (PC1/3 or PC2)	facilitates penile erection
(21, 34 **,** 35 **)**	AQEE-30	mass spectrometry in plasma, enzyme-linked immunosorbent assay in CSF and Gel chromatography	R585-A586 (PC1/3 or PC2)	facilitates penile erection, antidepressant-like effects, and prevents neuronal apoptosis
**(** 21, 36 **)**	LQEQ-19	matrix assisted laser desorption ionization-time of flight-mass spectrometry in rat brain	R596-L597 (PC1/3 or PC2)	prevents neuronal apoptosis; facilitates penile erection

Some VGF-derived peptides have been shown to have specific neuronal bioactivities (15) as shown in **Table 1**, for example, TLQP-62 induces transient potentiation in rat and mouse hippocampal slices (29, 37), increases synaptic activity (11), influences cognitive pathways (28), stimulates neurogenesis (30) and reduces neuronal death *in vitro* (33). Furthermore, Yamaguchi etal. (25) reported that water homeostasis is regulated by NERP-1 and NERP-2. TLQP-21 also regulates energy balance (12), gastrointestinal contractile activity (33), and has analgesic properties (38).

Proteomic studies that have investigated disease indicators have identified several peptides produced from the VGF precursor in CSF and cortex. These investigations identified a decrease in several VGF peptides in individuals with AD and PD (31, 39), including C-terminus peptides. Most studies have demonstrated a decrease in VGF-derived peptides in the CSF of people affected by AD (e.g., TLQP-62, VGF373-417, VGF64-80, VGF268-278), ALS (VGF398-411), and frontotemporal dementia (VGF26-62) compared to control (40–45). Furthermore, Cocco etal. (31) assessed VGF peptides in the cortex post-mortem and they observed a decrease in TPGH and NERP-1 peptides in people with PD and a decrease in C-terminus TPGH, NERP-1 and N-terminus in people with AD. Despite the difference in peptides evaluated between studies, the results showed a change in VGF-derived peptides in the CSF and cortex. It was therefore hypothesised that VGF and its derivatives could be potential biomarkers for diagnosing AD, PD, and ALS.

Thus, this study aimed to systematically search the literature to determine whether VGF and/or its derived peptides can be used as biomarkers for the diagnosis of AD, PD, and ALS. The objectives included (1) evaluation of the level of VGF and/or its derived peptides and (2) determination of the potential association between VGF and the accumulation of amyloid-beta, lack of dopamine, and cognitive decline.

## Methods

The Preferred Reporting Items for Systematic Reviews and Meta-Analyses (PRISMA) checklist was used to conduct this systematic review (46).

### Search strategy

A systematic search of the literature was performed from 14^th^ June 2021 to July 2021 by two independent researchers (S.A and M.A). Electronic databases (Ovid EMBASE, Cochrane Library, PubMed, Scopus, and Web of Science) were searched using the following terms: (“VGF” **OR** “VGF peptides” **OR** “VGF-Derived Peptide” **OR** “VGF-Derived Peptides” **OR** “NERP-1” **OR** “NERP-2” **OR** “neuroendocrine regulatory peptides 1” **OR** “neuroendocrine regulatory peptide 2” **OR** “TPGH” **OR** “TLQP-62” **OR** “TLQP-21” **OR** “AQEE-30” **OR** “AQEE-10” **OR** “HHPD-41” **OR** “LQEQ-19” **OR** “AQEE-11”) **AND** (“Neurodegeneration disease” **OR** “Neurodegenerative disease” **OR** “Parkinson’s disease” **OR** “Alzheimer’s disease” **OR** “dementia with Lewy bodies” **OR** “amyotrophic lateral sclerosis” **OR** “frontotemporal dementia”).

All identified studies were saved from the databases in EndNote, and duplicates were removed.

### Eligibility criteria

The current systematic review examined study eligibility using a population comparison and outcome, as shown in **Table 2**. There is no intervention because this systematic review aimed to investigate the level of VGF in adults with neurodegenerative diseases compared to adults without neurodegenerative diseases. Only full-text studies were included, regardless of publication date (there was no restriction on publication date).

- Study design: An observational study to address the research question.
- Population: Adults (humans) with neurodegeneration diseases (Parkinson’s disease, Alzheimer’s disease, dementia with Lewy bodies, amyotrophic lateral sclerosis, or frontotemporal dementia) compared to adults with normal cognitive function.
- Outcome: The primary outcome is the level of VGF and/or derived peptides.

**Table 2:** Population–intervention–comparison–outcome (PICOS) criteria for study inclusion and exclusion.

Criteria category	Inclusion	Exclusion
**Population**	Human adults (aged ≥ 18 y) with neurodegeneration diseases (Parkinson’s disease, Alzheimer’s disease, dementia with Lewy bodies, amyotrophic lateral sclerosis, or frontotemporal dementia). The reason for including young adults because ALS affects both young and old adults	Animal/children (age < 18 y) studies
**Intervention/exposure**	No intervention	None
**Comparators**	Adults without neurodegeneration diseases	Children/animals
**Outcome measure**	VGF and/or derived peptides	No analysis of VFG
**Study design**	Observational studies	Randomised control trials/review studies/case reports/pilot studies

### Exclusion criteria

- Review studies
- Case reports
- Intervention study
- Non-English studies
- A full-text report of the study was not available

### Data extraction and synthesis

The following data were extracted from all the studies included: the author’s name, the year of publication, study location, study design, the target population, the sample size, the average age, and the gender of the participants, education level, disease duration, medication, proteomic approach, type of VGF-derived peptides, the primary outcome, VGF/VGF-derived peptide levels and the secondary outcomes, Aβ1-42, cognitive score, and dopamine. Due to the heterogeneity of the data, a meta-analysis was not performed. For example, there was a difference in the type of VGF peptides evaluated in the studies and there were differences in participant characteristics and neurodegenerative diseases reported in the included studies.

### Quality assessment

Following data extraction, the study quality was evaluated by two researchers (M.A. and S.A.) using the National Heart, Lung, and Blood Institute (NHLBI) Quality Assessment Tool for Case-Control Studies (47). The tool comprises 12 questions (**Table 3**). These questions were answered with either yes, no, not report, or not applicable.

**Table 3:** Quality assessment.

Criteria
1. Was the research question or objective in this paper clearly stated and appropriate?
2. Was the study population clearly specified and defined?
3. Did the authors include a sample size justification?
4. Were controls selected or recruited from the same or similar population that gave rise to the cases (including the same timeframe)?
5. Were the definitions, inclusion and exclusion criteria, algorithms or processes used to identify or select cases and controls valid, reliable, and implemented consistently across all study participants?
6. Were the cases clearly defined and differentiated from controls?
7. If less than 100 percent of eligible cases and/or controls were selected for the study, were the cases and/or controls randomly selected from those eligible?
8. Was there use of concurrent controls?
9. Were the investigators able to confirm that the exposure/risk occurred prior to the development of the condition or event that defined a participant as a case?
10. Were the measures of exposure/risk clearly defined, valid, reliable, and implemented consistently (including the same time period) across all study participants?
11. Were the assessors of exposure/risk blinded to the case or control status of participants?
12. Were key potential confounding variables measured and adjusted statistically in the analyses? If matching was used, did the investigators account for matching during study analysis?

## Results

### Eligibility of studies

**Figure 2:**
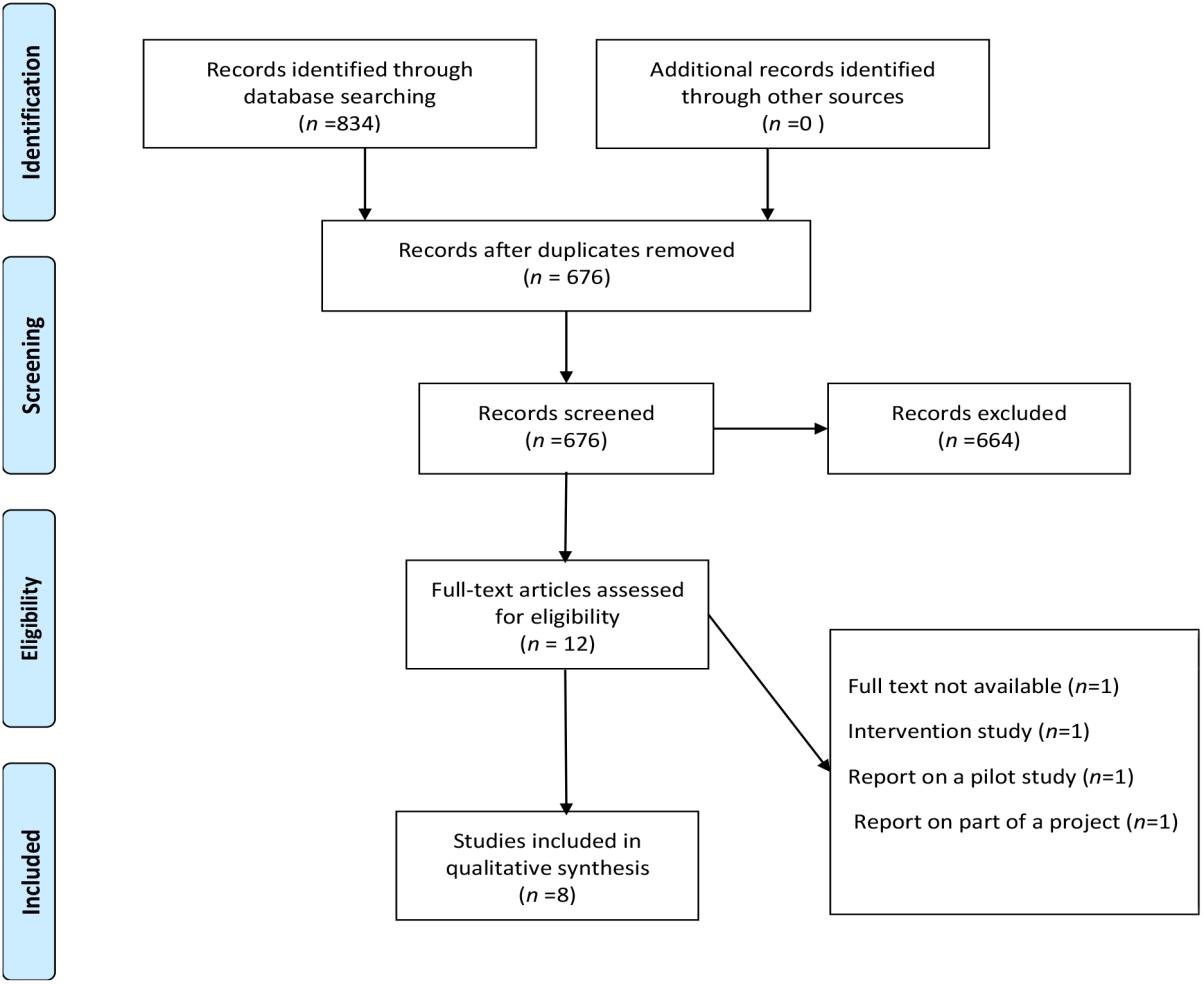
Stages of study selection.

Five databases (Ovid EMBASE, Cochrane Library, PubMed, Scopus, and Web of Science) retrieved 834 studies, of which 158 were duplicate studies, leaving 676 studies. Further screening of the title and abstract led to the exclusion of 664 studies, leaving 12 studies to be evaluated against the inclusion/exclusion criteria; of these, one was the full text not available, one was an intervention study, one was a pilot study, and one was part of a project. This left eight studies eligible for inclusion (**Figure 2**).

### Quality assessment

The quality of each study was evaluated using the NHLBI Quality Assessment Tool for Case-Control Studies (**Table 4**).

**Table 4:** NHLBI tool for quality assessment of the included studies.

Questions													
Study, Year	1	2	3	4	5	6	7	8	9	10	11	12	
Van Steenoven etal. (48)	 +	 +	 r	 +	 +	 +	 a	 r	 a	 a	 r	 +	
Llano et al. (49)	 +	 +	 r	 r	 +	 +	 a	 r	 a	 a	 r	 +	
Cocco etal. (31)	 +	 +	 r	 r	 r	 +	 a	 r	 a	 a	 r	 +	
Zhao etal. (45)	 +	 +	 r	 r	 +	 +	 a	 r	 a	 a	 r	 +	
Pasinetti etal. (42)	 +	 +	 r	 r	 r	 +	 a	 r	 a	 a	 r	 +	
Brancia etal. (27)	 +	 +	 r	 +	 r	 +	 a	 r	 a	 a	 r	 +	
Hölttä etal. (39)	 +	 +	 r	 r	 r	 +	 a	 r	 a	 a	 r	 +	
Cocco etal. (50)	 +	 +	 r	 +	 +	 +	 a	 r	 a	 a	 r	 +	


+, yes; 
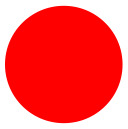
no; 
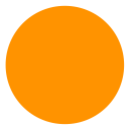
?, cannot determine; 

a, not applicable; 

r, not reported.

Overall, the studies reported on VGF changes in neurodegenerative diseases seem convergent and reliable enough to indicate distinct changes in the profile of VGF and VGF derived peptides in a range of neurodegenerative disease conditions.

### Study characteristics

The eight included studies involving a total of 673 individuals (441 adults with neurodegenerative disease and 232 adults without neurodegenerative disease) were published between 2006 to 2020. Three of the included studies were conducted in the USA (42, 45, 49);, three in Italy (27, 31, 50), one in Sweden (39), and one in the Netherlands (48).

Four studies were conducted on people with AD (31, 39, 48, 49), two studies were conducted on people with PD (31, 50), and three studies were conducted on people with ALS (27, 42, 45). The average ages of the participants were between 21.4 and 87.3 years, and the number of males was higher than females (348 vs. 241 respectively). However, one study did not mention the age and gender of the participants (45), and in the study conducted by Brancia etal. (27), the gender of the control group was not mentioned. The educational level of the participants in two studies was reported (48, 49), and the level of education was between 9 and 16 years.

Differences in the VGF-derived peptides were assessed in some of the included studies. These included VGF373-417 (48), VGF-NSEP (49), TPGH, NERP-1, C and N terminus peptide (31), C-terminus peptides ( 27, 45, 50), and 4.8-kDa VGF peptide (42).

Most studies used enzyme-linked immunosorbent assay (ELISA) to assess VGF peptides (27, 31, 45, 48, 50). Further, CSF samples were used in most of the studies (39, 42, 45, 48–50), except for two studies, one of which used plasma (27) and another used cortex (31).

The characteristics of the studies and results are shown in **Tables 5**, **6**. The secondary outcomes (Aβ1-42 and cognitive score) were assessed only in one study (48), and dopamine was used in only one study (50). The secondary outcomes are shown in **Table 7**.

**Table 5:** Characteristics of the included studies.

Author, Year	Study design	Location	ND/Controls	Female*n* (%)	Age,Years	Education, years	Type of VGF-derived peptides	Proteomic approach
Van Steenoven etal. (48)	Case control study	Amsterdam, Netherlands	DLB (*n* = 44)	5 (11%)	67±6	9-13	VGF VGF373-417	ELISA SRM (Using CSF samples)
			AD (*n* = 20)	2 (10%)	65±6			
			Controls (*n* = 22)	4 (18%)	63±5			
Llano et al. (49)	Case control study	USA	AD (*n* = 66)	117(40.9%)	75-76	15.1-16	VGF- NSEP	Mass Spectrometry (Using CSF samples)
			MCI (*n* = 135)					
			Controls (*n* = 86)					
Cocco etal. (31)	Case control study	Italy	PD (*n* = 6)	6(50%)	60-80	–	TPGH, NERP-1, C and N terminus	ELISA (Using cortex samples)
			AD (*n* = 5)	3(60%)	72-80			
			Controls (*n* = 8)	4(50%)	60-80			
Cocco etal. (50)	Case control study	Italy	PD (*n =* 63)	0	71.7	–	VGF C-terminus peptides	ELISA (Using plasma)
			Controls (*n* = 21)	0	67.9			
Zhao etal. (45)	Case control study	New York, USA	ALS (*n* = 17) Controls (*n* = 21)	–	–	–	C-terminus peptides	ELISA (Using CSF)
Pasinetti etal. (42)	Case control study	New York, USA	ALS (*n* = 36)	8 (22.22%)	21.4–87.3	–	4.8-kDa VGF peptide	SELDI-MS (Using CSF)
			Controls (*n* = 21)	8 (38%)	61–77			
Brancia etal. (27)	Case control study	Italy	ALS (*n* = 41)	18 (43.9%)	25–85	–	C-terminus peptides	ELISA (Using plasma)
			Controls (*n* = 45)	–				
Hölttä etal. (39)	Case control study	Sweden	AD (*n* = 8)	6 (81.25%)	72–81	**-**	VGF-derived Peptides	(LC−MS) (Using CSF)
			Controls (*n* = 8)	4 (50%)	51–72			

ND, neurodegenerative diseases; AD, Alzheimer’s disease; MCI, Mild cognitive impairment; PD, Parkinson’s disease; ALS= Amyotrophic lateral sclerosis; DLB, dementia with Lewy bodies; CSF, cerebrospinal fluid; LC−MS, Liquid chromatography coupled with mass spectrometry; ELISA, enzyme-linked immunosorbent assay; SRM, Selected reaction monitoring; SELDI-MS, Surface-enhanced laser desorption/ionisation time-of-flight mass spectrometry. (-), not available in the study

**Table 6:** Primary outcomes.

Author, Year	Results
Van Steenoven etal. (48)	VGF373-417 was lower in patients with DLB (2.5 pmol/mL) than those with AD (3.3 pmol/mL) and controls (3.6 pmol/mL) (*p <*0.05 and *p <*0.001, respectively). VGF in CSF was lower in patients with DLB (0.14 L.H. ratio) than in patients with AD (0.17 L.H. ratio) or controls (0.17 L.H. ratio), when assessed by SRM (*p <*0.01 and *p <*0.001, respectively)
Llano et al. (49)	CSF VGF levels were lower in the AD group compared to controls (*p* = 0.0002), with lower levels at baseline in MCI-AD converters than in non-converters (*p* = 0.032).
Cocco etal. (31)	In the PD samples, there was a significant decrease in TPGH compared to control (182.25 vs. 66.9 pmol/g, *p <*0.001, respectively) and NERP-1 peptides 3.89 ± 0.59 vs. 1.2 ± 0.1 pmol/g, p <0.01 controls vs. PD, respectively), while the other peptides did not change. In the AD samples, there was a significant decrease in all VGF peptides studied compared to the control, C-terminus (1330 ± 261 vs. 700 ± 120 pmol/g, p <0.045, controls vs. AD, respectively), TPGH (182 ± 25 vs. 68.2 ± 6.3, pmol/g, p < 0.010, controls vs. AD, respectively), NERP-1 (3.89 ± 0.59 vs. 0.8 ± 0.4, pmol/g, p <0.007, controls vs. AD, respectively), and N-terminus (10.7 ± 3.1 vs. 5 ± 1.1 pmol/g, p <0.002, controls vs. AD, respectively).
Cocco etal. (50)	Adults with ALS had less full-length VGF in their CSF compared to controls (*p <*0.05).
Zhao etal. (45)	The 4.8-kDa VGF peptide significantly low in adults with ALS compared to healthy controls (*p <*0.017).
Pasinetti etal. (42)	VGF C-terminus peptide levels were low in later stage ALS adults (*p <*0.04) compared to controls and early-stage ALS
Brancia etal. (27)	VGF peptides were significantly low in adults with AD compared to healthy controls (*p <*0.05)
Hölttä etal. (39)	VGF C-terminus peptide levels were lower in adults with PD at diagnosis compared to controls and patients on short-term dopamine replacement therapy (*p <*0.001)

**Table 7:** Secondary outcomes.

Author, Year	Results
Van Steenoven etal. (48)	**Aβ1-42/cognitive testing** ➢ VGF was positively associated with CSF tau (0.55 < r <0.75) but not with Aβ1-42 ➢ Lower VGF levels were linked to a more advanced stage of cognitive decline in DLB patients. Regarding AD patients, the association between VGF levels and cognitive tests was not discussed.
Cocco etal. (50)	**Dopamine** ➢ People with PD who used dopamine for over six years had a higher level of VGF C-terminus compared to those who did not use dopamine (*p <*0.001) or short-term dopamine use (*p <*0.05).

Aβ1-42, amyloid β1-42; p-tau, tau phosphorylated at threonine 181; t-tau, total tau; AD, Alzheimer’s disease; DLB, dementia with Lewy bodies.

### VGF and AD

Cocco etal. (31) investigated the levels of VGF peptides (TPGH, NERP-1, C-terminus, and N-terminus peptides) in post-mortem cortex samples of adults with AD compared to adults without neurodegenerative diseases. The results showed that in the AD samples, there was a significant decrease in all VGF peptides studied compared to the control; these included C-terminus (1330 ± 261 vs. 700 ± 120 pmol/g, *p <*0.045, controls vs. AD, respectively), TPGH (182 ± 25 vs. 68.2 ± 6.3, pmol/g, *p <*0.010, controls vs. AD, respectively), NERP-1 (3.89 ± 0.59 vs. 0.8 ± 0.4, pmol/g, *p <*0.007, controls vs. AD, respectively) and N-terminus (10.7 ± 3.1 vs. 5 ± 1.1 pmol/g, *p <*0.002, controls vs. AD, respectively). Similarly, Hölttä etal. (39) showed that the level of CSF VGF peptides was low in people with AD compared to healthy controls (*p <*0.05).

Furthermore, Llano et al. (49) showed that the level of VGF peptides was low in people with AD compared to healthy people (*p* = 0.0002), and Mild cognitive impairment (MCI)-AD converters had lower baseline levels than non-converters (*p* = 0.032). CSF A1-42, phosphorylated tau, hippocampal volume, and VGF peptide levels exceeded conventional biomarkers alone in predicting the conversion of MCI to AD.

However, Van Steenoven etal. (48) showed that the level of VGF373-417, as assessed by ELISA, was lower in people with dementia with Lewy bodies (DLB) (2.5 pmol/mL) than in those with AD (3.3 pmol/mL) or controls (3.6 pmol/mL) (*p <*0.05 and *p <*0.001, respectively). Similarly, levels of VGF in CSF were lower in people with DLB (0.14 L.H. ratio) than those with AD (0.17 L.H. ratio) or controls (0.17 L.H. ratio) when assessed by selected reaction monitoring (SRM) (*p <*0.01 and *p <*0.001, respectively). There was no significant difference in CSF VGF levels between people with AD and controls (*p >*0.05). Furthermore, VGF was positively associated with CSF tau (0.55 < r <0.75) but not with A1-42. There was also a relationship between cognitive tests and the level of VGF. Lower VGF levels were at a more advanced stage of cognitive decline at baseline, while higher VGF levels were correlated with a more severe longitudinal cognitive decline in DLB patients.

### VGF and PD

Cocco etal. (31) investigated the levels of VGF peptides in post-mortem cortex samples of adults with PD and found a significant decrease in TPGH compared to control (182. 25 vs. 66. 9 pmol/g, *p <*0.001, respectively) and NERP-1 peptides (3.89 ± 0.59 vs. 1.2 ± 0.1 pmol/g, *p <*0.01 controls vs. PD, respectively), while the other peptides did not change (C-terminus and N-terminus peptides). However, Cocco etal. (50) showed that VGF C-terminus peptide levels were lower in adults with PD at the time of the diagnosis (*n* = 23; no medication used) compared to control (*n* = 21) and people on short-term dopamine (1–6 years; *n* = 24) (*p <*0.001). People with PD who used dopamine for over six years had a higher level of VGF C-terminus compared to those who did not use dopamine (*p <*0.001) or had short-term dopamine use (*p <*0.05).

### VGF and ALS

Zhao etal. (45) showed that adults with ALS had lower amounts of full-length VGF in their CSF (*p <*0.05) compared to the control group, with a significant decrease in VGF in patients with weakness in two areas compared to only one area (*p <*0.05). Similarly, Pasinetti etal. (42) showed that a 4.8-kDa VGF peptide was significantly low in adults with ALS compared to healthy controls (*p <*0.017). However, Brancia etal. (27) performed a proteomic study to verify whether VGF peptides (C-terminal peptides of VGF) change in plasma in adults with ALS. The results demonstrated that the VGF C-terminus peptide level was lower in people in the later stage of ALS (*n* = 18) than those in the early stages of ALS (*n* = 23) and the control group (*n* = 45) (16% decrease, *p <*0.04).

## Discussion

Accelerated ageing is a global phenomenon with substantial consequences on health (6), as ageing is associated with neurodegenerative diseases (51), such as PD, ALS, and AD. However, there are no simple biomarkers that can predict the onset of these diseases. VGF and its peptides have recently been highlighted as potential biomarkers for the diagnosis of these diseases, therefore, this study systematically searched the literature to determine whether VGF and/or its derived peptide can be used as biomarkers for the diagnosis of PD, ALS, and AD. The analysis revealed that the use of VGF and its derivatives for the diagnosis of PD, ALS, and AD remains unclear.

### VGF and PD

Cocco etal. (31) showed that TPGH and NERP-1 peptides were reduced in the parietal cortex of adults with PD compared to healthy subjects (*p <*0.05) but other peptides such as C-terminus and N-terminus peptides did not change. By contrast, another study found a change in the C-terminus peptides in the plasma of people with PD at the time of the diagnosis compared to controls and people with PD on short-term dopamine replacement therapy (*p* < 0.001) (50). However, people with PD who used dopamine for the long term (>6 years) had higher levels of VGF (50). VGF C-peptide decreased in the substantia nigra of the 6-OHDA rat model according to the degree of dopaminergic neuron degeneration (50). This is consistent as pointed out in the present review that demonstrated a relationship between dopamine and VGF levels (50). Interestingly, dopamine release is increased by neurotrophic factors, NGF, in methyl-phenyl-tetrahydropyridine (MPTP)- treated mice (52), which in turn leads to the expression of VGF (53). However, Tokizane etal. (54) showed that VGF was increased in normal animals treated with sulpiride (dopamine antagonist), while it dropped in continuously stressed animals treated with bromocriptine (D-2 dopamine agonist). These results indicate that dopaminergic neurons negatively regulate VGF expression under sustained stress. This could be clarified by the cAMP signalling pathway, in which a cAMP-responsive element regulates VGF expression (55). Thus, the dopamine inhibition may lead to cAMP build-up and later expression of VGF mRNA.

### VGF and ALS

Pasinetti etal. (42) reported that the 4.8-kDa VGF peptide was significantly low in adults with ALS compared to healthy controls using CSF samples (*p <*0.017). Furthermore, Zhao etal. (45) observed that adults with ALS had lower amounts of full-length VGF (C-terminus peptides) in their CSF compared to controls (*p <*0.05), and the lower the level of VGF, the greater the muscle weakness. However, Brancia etal. (27) reported that plasma VGF content was reduced only in the late stages of ALS, whereas the above-mentioned CSF studies found a decline in the early stages of ALS. Consequently, it appears that a decrease in VGF occurs first in the CSF and then in the plasma. Indeed, VGF peptides in the plasma can originate from the CSF, which is in close contact with the extracellular space of the brain and reflects metabolic changes. The levels of VGF peptides and their sequences in the human brain and CSF can help interpret and validate changes seen in patient plasma. Similarly, in animals, the loss of full-length VGF in the CSF, motor neurons, serum, and spinal cord was observed in asymptomatic G93A-SOD1 transgenic mice (~75 days), showing a gradual decrease as the animals weakened (45). However, decreased plasma VGF was observed in the late stages of ALS in mice (27), not in the early stages of ALS, as observed in the G93A-SOD1 mice (45). This could be explained by the antiserum raised against AQEE-30 (45) versus the antisera raised against a nonapeptide covering the C-terminus in the Brancia etal. (27) study.

### VGF and AD

Furthermore, changes and differences in VGF and/or its derived peptides were parallel in most studies. Three studies showed a significant decrease in the VGF-derived peptide level in people with AD compared to healthy controls (31, 39, 49). Although Van Stenoven etal. (48) observed that the VGF levels were theoretically low in adults with AD compared to healthy control, these variations did not achieve statistical significance (*p >*0.05), possibly due to the small sample size (*n* = 20) and wide variation within the AD group. Another possibility is that VGF-derived peptides have diverse responses and changes in different states. A recent study conducted by Beckmann etal. (56) showed that VGF is a critical regulator of AD, according to multiscale causal networks. Overexpression of VGF improves cognitive function and neurogenesis in 5xFAD mice (56), but low levels of VGF have been linked to cognitive deficits among people with DLB (48). VGF-derived peptides (TLQP-62) control neuronal activity, memory formation, and progenitor proliferation in the hippocampus through BDNF/TrkB signalling (37, 57). Furthermore, Cero etal. (58) reported that TLQP 21 activates the complement 3a receptor (C3aR1), consequently, C3a activation of C3aR1 regulates the uptake of amyloid on the microglia (34). Indeed, Van Steenoven etal. (48) showed that VGF is negatively associated with Aβ1-42, indicating the potential role of VGF in the pathophysiology of AD. In addition, VGF may be a therapeutic target for people with AD. A recent study showed that amyloid plaques was decreased in mice treated with TLQP 21 (59). However, further investigations are needed to determine the effects of TLQP 21 in humans.

### Potential mechanism of VGF

The mechanism underlying the VGF changes in neurodegenerative diseases is not yet clear, however, there is no doubt that neurotrophic factors, such as BDNF, are important for neuronal growth, survival, and differentiation (60). Furthermore, BDNF has a role in learning and memory by attaching to the TrkB receptor in the hippocampus, cortex, and basal forebrain (61). The level of BDNF mRNA expression in the hippocampus among people with AD is low (62). Similarly, a post-mortem examination revealed that people with AD had a low level of BDNF in their brain tissue (63, 64). A low level of BDNF has also been observed in the brains of people with AD, PD, and Huntington’s disease (65). The lack of BDNF in mice has been shown to enhance cognitive deficits (66). Therefore, it can be assumed that the decrease in VGF may be related to the loss of neuronal products, such as BDNF, which leads to a failure of neuroprotective functions. Vitamin D may contribute to the pathophysiology of neurodegenerative diseases and the mechanism of action of VGF, for example, low serum or plasma vitamin D levels have been linked to an increased risk of dementia (67, 68), and loss of balance (69), symptoms of neurodegenerative diseases. In aged rats, vitamin D3 prevents cognitive loss and improves hippocampal synaptic activity (70), suggesting a causal relationship between vitamin D levels and cognitive function. Indeed, Latimer etal. (70) showed that optimum levels of vitamin D3 play a role in cognitive processes by stabilizing myelin structure and enhancing transcription factors and synaptic vesicle recycling, indicating that vitamin D3 may improve the likelihood of brain ageing. Furthermore, Lewis etal. (71) demonstrated that in both undifferentiated and differentiated SH-SY5Y cells, vitamin D3 greatly enhanced VGF endogenous mRNA expression, suggesting that vitamin D3 may be a regulator of VGF. Interestingly, both Brewer etal. (72) and Oermann etal. (73) demonstrated that vitamin D3 plays a role in enhancing neuroprotection. Furthermore, the proliferation of the SH-SY5Y cell line is inhibited when it is treated with vitamin D3 (74). Nevertheless, long-term incubation with vitamin D3 produces only a minor differentiation tendency (74), hence, further investigations are needed to determine the effects of vitamin D3 in humans.

## Strength and limitation

The strength of this systematic review is using five databases to search for studies related to the research question. Furthermore, the quality of all studies was evaluated using the NHLBI Quality Assessment Tool for Case-Control Studies. However, published studies on VGF and neurodegenerative diseases are limited; therefore, this systematic review only involved eight studies, all of which were observational, indicating a high risk of bias. Furthermore, there was a significant difference between the included studies. For instance, there were differences in the types of peptides derived from VGF in studies, the types of neurodegenerative diseases studied, and the methods used to measure VGF in various samples—some studies used plasma, while others used CSF or cortex post-mortem. Lastly, most studies did not report participant characteristics, such as medications used and cognitive testing.

## Conclusion

The current systematic review discussed whether VGF and/or its derived peptide can be used as biomarkers for the diagnosis of amyotrophic lateral sclerosis, Parkinson’s disease, and Alzheimer’s disease. The findings showed that changes and differences in VGF and/or its derived peptides were parallel in most studies. VGF levels were positively correlated with those of tissue dopamine but not with Aβ1-42. Furthermore, low levels of VGF were associated to the cognitive deficits. These findings indicate the need for further investigation of the role of VGF in neurodegenerative diseases and pathophysiology to provide new insights.

## Data availability statement

The original contributions presented in the study are included in the article/Supplementary Material. Further inquiries can be directed to the corresponding author.

## Author contributions

Methodology; SA, MA; formal analysis SA, MA; investigation SA, MA; writing—original draft preparation SA; writing—review and editing SA. All authors contributed to the article and approved the submitted version.
